# Effects of Social Capital on General Health Status

**DOI:** 10.5539/gjhs.v6n3p45

**Published:** 2014-02-14

**Authors:** Ayano Yamaguchi

**Affiliations:** 1 College of Foreign Studies, Reitaku University, Kashiwa, Chiba, Japan

**Keywords:** social capital, health status, income inequality, social relationships, psychosocial factors, trust, social networks

## Abstract

This paper discusses the concept of social capital as a potential factor in understanding the controversial relationship between income inequality and individual health status, arguing a positive, important role for social capital. Most of the health research literature focuses on individual health status and reveals that social capital increases individual health. However, the difficulty in measuring social capital, together with what may be the nearly impossible task of attributing causality, should relegate the concept to a more theoretical role in health research. Nonetheless, social capital receives academic attention as a potentially important factor in health research. This paper finds that the mixed results of empirical research on income inequality and health status remain a problem in the context of defining a stable relationship between socioeconomic status and health status. Clearly, further research is needed to elaborate on the income inequality and health relationship. In addition, focused, rigorous examination of social capital in a health context is needed before health researchers can comfortably introduce it as a concept of influence or significance.

## 1. Introduction

Findings from previous studies on income and health have been extensively discussed in health literature (e.g., MacIntyre et al., 1998; Marmot et al., 1984; Adler et al., 1994). A positive relationship between socioeconomic status (SES) and individual health status remains an important factor in maintaining good health. However, previous studies have also discussed the relatively mixed findings on the actual relationship between income inequality and individual health status (e.g., Kawachi et al., 2000; Grossman, 1972; Soonbader et al., 1999).

For better understanding of such varied findings among individual-SES and income-inequality studies, social capital has been introduced as a possibly related concept. Researchers have considered using social capital as a means of understanding how income inequality might be associated with health disparities (Kawachi et al., 1997a). The concept of social capital provides an interesting perspective for integrating existing knowledge about social factors into health issues; additionally, social capital can further our understanding of how social conditions matter for health (Berkman et al., 2000).

Recently, researchers have paid increasing attention to social capital as an explanatory factor of individual environmental characteristics (Kawachi et al., 1999; Rose, 2000). Since a few studies have explored the relationship between social capital and individual health status, in this paper, I discuss issues pertaining to social capital and individual health status and, necessarily, the varied definitions of social capital. Initially, I provide the historical background on the concept of social capital. Contemporary sociological researchers found this concept appealing and hence, conducted research to measure it empirically (Putnam, 2000). Next, I discuss the origins, contemporary conceptualizations, empirical study applications, and critiques of social capital in relation to individual health status. Finally, I explore a theoretical perspective of the impact of social capital on individual health status; based on these findings, I suggest certain theoretical and practical contributions of this paper. Social capital is a controversial issue, and no consensus exists in health research literature on its definition and measurement; thus, social capital is a subject that may reward further investigation. This paper attempts to develop a theoretical perspective on social capital that, while at least partially addressing definition and measurement issues, more clearly addresses individual health status.

### 1.1 Socioeconomic Status as a Determinant of Health

Socioeconomic status (SES) is defined as the position of individuals within a socially stratified system. SES is a way to measure what people need in order to obtain desired outcomes or goals (House et al., 2000). In particular, the higher one’s SES, the greater one’s chances for a longer and better life. This positive relationship has been found consistently for populations across location (Robert, 1999) and historical period (Antonovsky, 1967).

Conversely, the lower one’s SES, the more negative relationships exist between various SES indicators. For example; income, education, employment status (Sorlie et al., 1995), occupational class or level (MacIntyre et al., 1998); and a wide range of acute, infectious, and chronic health conditions; and actual mortality (Adler et al., 1994; Marmot et al., 1984). Moreover, the relationship between SES and health outcomes exists not only at the “bottom rung” of the social-class “ladder,” but also at every individual SES rung. In other words, those at the rung just above the poverty line have a better general health status than those just below it; those at the top rung enjoy a better general health status than those just one rung below them (Adler et al., 1994). Not surprisingly then, many different perspectives and approaches were proposed to explain the relationship between SES and health status, and most of the research has focused on the effects of individual SES and income inequality on general health.

### 1.2 Income Inequality and Health

Researchers observed the individual to understand how SES may impact health. For instance, Grossman (1972) examined the idea that the individual produces his or her own health and benefits by confounding market goods and his or her own time. Health-production functions are analogues to this argument: health is thus specified as a function of the individual’s characteristics (Grossman, 1972), with little or no attention paid to relative social position. Preston (1975) suggested that additional income impacts health as measured by mortality, more positively in those with low income than those with high income.

A number of studies have explored the absolute income hypothesis that posits health increases according to the income of the society in which the individual resides. This contrasts with the relative income hypothesis that posits health increases with an individual’s income relative to others in the society. Regarding the relative income hypothesis, Kawachi et al. (2000) explained that

*…health depends not just on one’s own level of income, but also on the incomes of others in society. At any given level of income, the hypothesis states that an individual’s health status depends on the rank within the income distribution that is bestowed upon the individual by his or her level of income, and/or the distance between his or her income and the average income (p. 649)*.

Directly testing the relative income hypothesis is difficult “because of the lack of agreement about the appropriate reference group for social comparison. An indirect test of the relative income hypothesis is offered by examining the relationship between income distribution and individual health” (Kawachi et al., 2000, p. 649).

Research on income inequality and health outcomes has yielded mixed results. Some studies suggested the effect of income inequality on health behaviors, self-rated general health status, and individual mortality. In addition, further studies replicated such results. However, others revealed a differential effect of income inequality on income groups after controlling for individual income (Kawachi et al., 2000).

Furthermore, some researchers have pointed out that “the practice of adjusting for individual income when finding an effect of income inequality has its own problems” (Kawachi et al., 2000, p. 650). Diez-Roux et al. (2000) mentioned that

*…the analytical separation of these two mechanisms (i.e., the effects of absolute and relative differences) may be theoretically interesting but is also artificial, because both are inextricably linked. In reality, adjusting inequality effects for individual-level income necessarily leads to an underestimation of the total inequality effect on health (p. 685)*.

However, overall, researchers are more likely to address the mechanisms and operations behind the link between income inequality and health outcomes (including general health status). Some research in this direction has emphasized the psychosocial damage inherent to social comparisons and relationships within an unequal society. However, other studies have emphasized the indicators and patterns of social relationships and investments that suggest a growing distance between the rich and the poor (Kawachi et al., 2000, p. 650). Moreover, psychosocial interpretations help to explain mechanisms and operations that potentially link income inequality and health.

Psychosocial interpretation has emphasized relative deprivation, popularized as a social determinant of health by Richard Wilkinson (1997). Wilkinson’s work is central to discussions on the value of studying not only income but also income *inequality*. In other words, health disparities due to SES are related to individuals’ SES placement relative to one another. Wilkinson (1997) observed that income inequality impacts health more negatively in locations with wide income disparities. In theory, income inequality impacts health via perceptions of one’s place in the hierarchy in two ways (Wilkinson, 1997). Internally, a negative perception of one’s place gives rise to negative emotions that impact health via psycho-neuroendocrine mechanisms and unhealthy behaviors. Externally, such negative emotions foster anti-social behavior, for instance, a decrease in social engagement. On the individual level, such a withdrawal may impact health via a variety of social mechanisms, including isolation, mistrust, insecurity, and lowered self-esteem. On a societal level, withdrawal may lead to decreased social cohesion and social capital, both linked to poorer population health outcomes.

Since the findings supporting relative deprivation originate from animal studies (Adler et al., 1994), this theory has been difficult in applying to explanations of income inequality and health status. Nevertheless, Wilkinson (1997) supported the argument through three findings: First, overall mortality in developed countries more closely relates to relative income within countries than to differences in absolute income between countries. Second, national mortality rates tend to be lower in countries with smaller income discrepancies and thus, lower levels of relative deprivation. Third, a rise in life expectancy appears unrelated to economic growth rates. Thus, although material and social influences contribute to health disparities, the significance of *relative SES* seems to confirm psychosocial influences (Wilkinson, 1997).

Particularly, Wilkinson (1997) stated that in a sample of 23 member countries of the Organization of Economic Cooperation and Development (OECD) in 1993, absolute income (measured via the gross domestic product or GDP) was not related to life expectancy. However, Lynch et al. (2000) challenged Wilkinson’s psychosocial interpretation by analyzing these same variables for 155 OECD member countries. Interestingly, Wilkinson had not thoroughly described his sample-selection method. Lynch and colleagues’ r-correlation for their complete sample was much larger than Wilkinson’s and statistically significant. Moreover, unlike the data from the 23 countries Wilkinson sampled (all with a GDP per person greater than $10,000), their data showed a positive linear relationship for all countries above that GDP per person threshold. Therefore, Lynch and colleagues concluded that the relationship between absolute income and life expectancy among wealthier nations is affected by the choice of countries included. Thus, they challenged the validity of a critical finding that Wilkinson used to support his theory. Obviously, we need in-depth research, particularly extending beyond national and cross-national ecologic samples, prior to a truly meaningful discussion for or against relative deprivation.

Consequent to the findings summarized above, the issue of income inequality and its effect on health status is controversial, and therefore, we turn to social capital for answers. I hypothesize that social capital mediates the relationship between income inequality and individual health status.

### 1.3 Definitions of Social Capital

Social capital has a rather long history in the social sciences. Lyda Hanifan (1920) first used the term in the contemporary sociological sense: she defined social capital as the role of community participation in sharing local educational outcomes. Later in the century, Jane Jacobs (1961), Glenn Loury (1977), and James Coleman (1988) continuously developed social capital as a concept. A French sociologist, Pierre Bourdieu (1983, 1986) has developed a parallel explanation and defined social capital in positive relation to community and governance in Italy in the 1990s. In contrast to his observations of Italy, he asserted that Americans in the late 20^th^ century are “bowling alone” (Woolcock, 1998b).

Throughout its history, social capital has taken various forms. Paxton (1999) defined a physical social capital concept as the ways that implements, such as tools or machines, are used. Thus, social capital facilitates and promotes the production of physical capital. Becker (1964), building on Schultz’s work (1961), presented the concept of human capital and argued that individuals, through education or job training, hold within themselves the ability to facilitate production. This later concept of social capital, comprised of implements and individuals, indicates that certain social relations facilitate production (Paxton, 1999).

When defining social capital as the resources that result from social structures, Bourdieu et al. (1992) is often quoted as “Social capital is the sum of the resources, actual or virtual, that accrue to an individual or group by virtue of possessing a durable network of more or less institutionalized relationships of mutual acquaintance and recognition” (p. 119). Coleman clarified (1988, 1990) social capital as a function of social structure producing advantage:

*Social capital is defined by its function. It is not a single entity but a variety of different entities having two characteristics in common. They all consist of some aspect of a social structure, and they facilitate certain actions of individuals who are within the structure (Coleman, 1988, S98)*.

Grounding his influential work in that of Coleman, although Putnam (1995) preserved the focus on action facilitated by social structure, he added that networks within social capital play an important role in the provision of other aspects of social capital. The most cited in health research, Putnam defined social capital as “features of social organization, such as networks, norms, and social trust that facilitate coordination and cooperation for mutual benefit” (p. 67). On the basis of his research on civic traditions and local government in Italy (*Making Democracy Work*) (Putnam, 1993). Putnam (2000) conceptualized social capital by incorporating Alexis de Tocqueville’s ideas of associational life being important for nations. Specifically, the amount of social capital in a community—town, state, or nation—is a collective characteristic generated through norms of reciprocity and trustworthiness among residents, and has implications for a multitude of beneficial outcomes for that community.

Lin (1999) defined social capital as access to and use of resources embedded in social networks. Lin’s premise of social capital is rather simple and straightforward: “investment in social relations with expected returns” (p. 30). Social capital consists of “individuals engage[d] in interactions and networking in order to produce profits” (p. 31).

Portes et al. (1993) explained that introducing the concept of social capital reinforced sociological perspectives. Although, he clarified social capital as the umbrella concept of social “embeddedness,” this raises a question about the “arena” of civic society. As envisioned by Coleman and Bourdieu, Woolcock (1998a; 1998b) most systematically integrated and unified a broad “arena”; in Putnam’s footsteps, he included the possibility of studying social capital at different societal levels.

In contrast to the others, however, Woolcock is more likely to apply social capital concepts to analysis of national and community development in Third World countries. In the conceptualization of social capital itself, he suggested four dimensions. The first two dimensions are distinct but complementary, based on the concepts of “embeddedness” and “autonomy.” The first concept, embeddedness, supports and follows Granovetter’s (1985) contribution that posits all economic action to be inherently enmeshed in social relations of one configuration or another, and development essentially brings about a change in the kind, not degree, of embeddedness. Taking this a step further, however, Woolcock claimed that the senses and tones in which social embeddedness and autonomy are involved at the micro and macro levels are not the same: embeddedness at the micro level refers to intra-community ties; whereas, at the macro level, it relates to state-society relations. Autonomy at the micro level links to extra-community networks; however, at the macro level, it links to institutional capacity and credibility. Thus, Woolcock (1998a) noted that any synthesis of social capital as it has developed at the micro macro levels may have to integrate or unify four distinct dimensions. He referred to embeddedness at the micro level as “integration” and autonomy at the macro level as “organizational integrity.” Woolcock stated that combinations of these four dimensions—embeddedness, autonomy, integration, and organizational integrity—can account for a range of development outcomes.

Although researchers provided many definitions of social capital, all the definitions seem to be relational and multidimensional. However, researchers also provided an explicit contrast between the two types of definition. First, social capital is considered an aggregate of the individual (Bourdieu, 1983, 1986; Bourdieu et al., 1992; Coleman, 1988, 1990). Second, it is the notion of resources collectively possessed (Putnam, 1995, 2000).

### 1.4 Critiques of Social Capital in Empirical Studies

Baron, Field, and Schuller (2000) suggested “over versatility” as a major criticism of social capital. However, over versatility relates more to its various applications than to its intrinsic quality as a concept. In addition, these researchers suggested that societies cannot be sufficiently understood through study of the individual, and they shift analytical focus from behavior of individual agents to social patterns among agents, social units, and institutions.

Arrow (2000) explained the lack of consensus for adding “social capital” to other forms of capital, and Solow (2000) clarified that, so far, social capital is only “seen vague ideas and causal empiricism” (p. 6). There is no value added with this conceptual framework, despite the intentions of those who study social capital to understand the interaction between society’s institutions and their shared attitudes about the way the economy works (Arrow, 2000; Solow, 2000).

Labonte (1999) suggested that social capital may be just a fad in the social sciences, wondering if it is a “‘Trojan horse’ for colonization from any side of the ideological spectrum.” The current notions of social capital reveal an element of repackaging in the literature. However, the same can be said of any theoretical construction (Labonte, 1999).

Almedom (2005) pointed out that social capital is a complex, compound construct. It can be both an asset and a liability to receivers and providers of services and other interventions. In measurement, the most meaningful assessment of social capital may examine individual *access* or *process*, rather than *possession*. After all, social capital is a property belonging to groups and therefore, an economic variable.

Baum (1999) and Labonte (1999) suggested that increasing social capital may negatively affect government support for social services or agencies because social capital supports individuals in the community, thereby reducing the burden on government. Governmental officials, administrators, and researchers need to realize that the purpose of social capital is not to provide the state with an excuse for nonsupport, but to maintain quality of life in the community.

Portes (1998) identified positive and negative aspects of social capital, its function and operation in community and society. First, those with higher levels of social capital are more likely to receive benefits in certain environments, thus increasing social control. A second benefit is that social capital produces the familial and social support that primarily benefits children. Familial social capital is embodied in the parent–child relationship; when parents are an important part of their children’s lives, the children’s intellectual development and socialization improves (Coleman, 1988). Third, social capital occurs through extra-familial networks, where ties and associations with other individuals and groups can help people gain direct access to economic resources and valued credentials.

For the negative consequences that Portes (1998) identified, the first is exclusion of outsiders. The second is that social group or social community closure may reveal the success of its members’ business initiatives. Third, social capital may restrict individual freedom and autonomy. The fourth negative consequence is that social capital tends to be a downward-leveling norm. In some social situations, group solidarity is produced by a common experience of adversity wherein individual success stories of group members may lead to cohesion. As a result of these observations, Portes pointed out that social capital may negatively influence individual lives, because the negative influences may need more material to be convincing.

Recently, Edmondson (2003) suggested that social capital serves as a critique of communities. Currently, social capital is viewed as a source of support for good health even though some argued that the concept should not be used to create neutral policy strategies. Edmondson questioned whether social capital is essential for moral and political debate on health policy.

### 1.5 Dimensions of Social Capital

Somewhat similar to Woolcock, Putnam (2000) presented certain dimensions of social capital—bonding, bridging, and linking social capital. Bonding social capital denotes the social relations among relatively homogeneous groups (e.g., ethnic, religious, or socioeconomic); it strengthens social bonds within a particular group. Bridging social capital constitutes the social relations among heterogeneous groups, and it strengthens ties across such groups. Linking social capital denotes the social relations between individuals and groups in a stratified hierarchy where different groups access power, social status, and wealth. Putnam (2000, pp. 22–23) noted as follows:

Bonding social capital is good for undergirding specific reciprocity and mobilizing solidarity. Dense networks in ethnic enclaves, for example, provide crucial social and psychological support for less fortunate members of their community, while furnishing start-up financing, markets, and reliable labor for local entrepreneurs. Bridging networks by contrast, are better for linkage to external assets and for information diffusion. Economic sociologist Mark Granovetter has pointed out that when seeking jobs—or political allies—the “weak” ties that link me to distant acquaintances who move in different circles from mine are actually more valuable than the “strong” ties that link me to relatives and intimate friends whose sociological niche is very like my own. …Bonding social capital constitutes a kind of sociological superglue, whereas bridging social capital provides a sociological WD-40.

Although much health research has employed Putnam’s conceptualization of social capital, it does not directly address health issues. Therefore, James et al. (2001) offered a conceptual framework for social capital and health status. Although they accepted Putnam’s conceptualization of social capital at the community level, they viewed health from a different perspective. They defined social capital as “resources in social relationships” that include “mutual trust, a sense of reciprocal obligation, and civic participation aimed at benefiting the group or community as a whole” (pp. 165–88).

On the basis of Putnam’s dimensions of social capital, Woolcock (2001) developed three forms: bonding, bridging, and linking. Bonding social capital denotes social connections among strong ties. It involves a high level of trust in a “network of people individuals turn to when individuals are sick or need an important errand run” (p. 13). Bridging social capital unifies people “who share broadly similar demographic characteristics.” Bridging is characterized by the “building of connections between heterogeneous groups” and the connections that are “likely to be more fragile but foster social inclusion” (p. 13). Linking social capital constitutes social connections with people in positions of power, whether political or financial. Linking relates to connections in the civic community to the political and financial environment, and it relates “to the capacity to level resources, idea[s], and information from formal institutions beyond community” (p. 13).

### 1.6 Contemporary Development of Social Capital in Health Research

Social capital has been proposed as a leverage point for understanding the literature’s collective findings on individual SES and income inequality. Several social epidemiologists (Kawachi et al., 1997a; Berkman et al., 2000; Kawachi et al., 1999; Rose, 2000) have received credit for introducing social capital to public health and have considered its utility for understanding routes through which income inequality is associated with health disparities (Kawachi et al., 1997a).

With a multitude of studies supporting the importance of social adhesion for health, social capital offers an interesting way to gain understanding of how social conditions influence health (Berkman et al., 2000). Therefore, the public health arena has seen a rapid emergence of research on social capital (Kawachiet al., 1999; Rose, 2000). However, the role of social capital in understanding how social conditions impact health means different things to different researchers. In other words, the routes through which social capital is associated with health are hypothesized differently, according to varied psychosocial interpretations. Researchers studying the psychosocial interpretation of income inequality on health have tended to view social capital as a promoting route (Wilkinson, 1997; Kawachi et al., 1997b).

### 1.7 Measurements of Social Capital

For empirical measurement, the myriad definitions and theories of social capital are problematic. The problem worsens as researchers try to create valid measures of social capital because the theoretical conceptualizations and frameworks actually drive the choice of research measures.

In clarifying the linkage problem from theoretical conceptualizations to measurement, Cattel (2001) explained that empirical studies have not succeeded in fully addressing the construct’s complex nature, particularly in contextualizing the place-based issue of social capital within specific localities or areas of analysis. In other words, social capital measured at the state or national level is very different from social capital measured at the neighborhood, community, or even non-place-based social network level. While theoretical arguments can be made for social capital should to be considered at each of these levels, such justification needs to be made explicit.

Health researchers have yet to explore adequately how different forms of social capital (e.g., social support, social leverage, informal social control, and participation in neighborhood organizations) may contribute to different outcomes. Using questions from the U.S. General Social Survey (GSS), Putnam (1995) explored the role of social capital, for instance, in networks, norms, and trust for citizen engagement in community affairs. Moreover, the questions from GSS were used for several epidemiological studies. While studying poverty and social capital in Tanzania, Narayan (1997) developed a Social Capital Index inspired in part by Putnam’s work in Italy. In Narayan’s survey, the thrust of which was defined as “personal belonging,” the householders responded according to three dimensions of social capital: their membership in groups, the characteristics of those groups, and individual values and attitudes. That Narayan’s study does not include information on reliability or validity serves, perhaps, to underline the difficulty of such measurement.

Research on children who prosper in unfavorable environments (Runyan et al., 1998) provided an example of social capital measurement without proper construct definition. These researchers presented a broad, brief definition of social capital and directly proceeded to create an index, or measurement, of social capital, using a 0−5 point scale. The items measured were the following, whether: two parents resided in the home; social support was provided for the primary caregiver; more than two children resided in the home; neighborhood support was provided; and the maternal respondent attended church or religious services. Unfortunately, no clear linkage exists between these measures and social capital.

Similarly, the measures that Gooden (1998) used to explore the health of rural African Americans in central Virginia appear questionable. Gooden (1998) chose the following to measure social capital: frequency of church attendance; community organization membership; employment outside of home; marital status; and telephone service in home.

Rose (2000) examined the relationship between social capital and individual health status by using a special-purpose questionnaire designed to measure social capital in a multiplicity of forms. The New Russia Barometer was employed to conduct a full-scale, multi-stage, randomly stratified sample covering the whole of the Russian Federation, urban and rural. For example, 1904 Russian respondents, aged 18 or over, were interviewed face to face in 191 widely dispersed primary sampling units. Aside from indicators of human capital, social capital measurements included social integration; and an individual’s cumulative use of networks and situation specific networks.

Finally, with the aim of providing a brief guide to operationalization and measurement, Lochner et al. (1999) reviewed the concept of social capital and its related constructs. They concluded that despite lacking a single definition of social capital and differing approaches to measurement, there appears to be agreement that community characteristics should be distinguished from individual characteristics and measured at the community level.

## 2. Conclusion

This paper discussed controversial relationships between income inequality and individual health status. For better understanding of differing study results on income inequality and individual health status, social capital was introduced as a potential mediating factor.

However, the many definitions of social capital currently under discussion make it difficult to determine its role in linking income inequality and health. All the definitions do seem to be relational and multidimensional, and researchers tend to divide the concept into two types. Social capital is first defined as an aggregate of the individual (Bourdieu, 1983, 1986; Bourdieu et al., 1992; Coleman, 1988, 1990) and, second, as resources collectively possessed (Putnam, 1995, 2000).

However, the health-status literature does illuminate an important, positive role for social capital, indicating that it helps to improve individual health status. Nevertheless, growing attention to the many and varied definitions has introduced levels of uncertainty about the use of social capital at the individual level, particularly in relation to health. The difficulty in measuring the concept, together with what may be the nearly impossible task of attributing causality, has relegated social capital to a more theoretical role in health research until such time as measurement and operational definitions attain consensus and more common use. Nonetheless, social capital continues to attract and receive academic attention as a potentially important factor in health research.

The lack of clarity in the relationship between income inequality and health status remains. Till now, credible theoretical and empirical work has been insufficient to offer social capital as a clarifier of these two other correlates. Until such time as a definitive standard exists for social capital and its measurement, the concept remains but a promising theory for health research. To improve our understanding of the mixed results of the income inequality and health status relationship, other, more mature, concepts and variables need to be used.

Clearly, further research is needed to elaborate on the income inequality and health relationship. In addition, focused, rigorous examination of social capital in a health context needed for before researchers can comfortably introduce it as a concept of influence or significance.

## 3. Implications

This paper suggests that social capital can indeed operate in the health status of various individuals and neighborhoods. It is clear that social capital is important for maintaining people’s general health status. In other words, despite gaps in social capital application, a wide range of community associations can improve individuals’ health status through outreach efforts that encourage development and maintenance of social integration and ties (Coleman, 1988; Putnam, 1995). For future empirical approaches, research must examine cross-cultural similarities and differences in individual behaviors in order to optimize the concept of social capital toward society’s benefit.

## References

[ref1] Adler N. E, Boyce T, Chesney M. A, Cohen S, Folkman S, Kahn R. L (1994). Socioeconomic status and health: The challenge of the gradient. American Psychologist.

[ref2] Almedom A. M (2005). Social capital and mental health: An interdisciplinary review of primary evidence. Social Science and Medicine.

[ref3] Antonovsky A (1967). Social class, life expectancy and overall mortality: Milbank Memorial Fund Quarterly. http://dx.doi.org/10.2307/3348839.

[ref4] Arrow K. J, Dasgupta P, Serageldin I (2000). Observations on Social Capital. Social Capital: A Multifaceted Perspective.

[ref5] Becker G. S (1964). Human capital: A theoretical and empirical analysis with special reference to education.

[ref6] Berkman L. F, Glass T, Brissette I, Seeman T. E (2000). From social integration to health: Durkheim in new millennium. Social Science & Medicine.

[ref7] Bourdieu P, Richardson J (1983). The forms of capital. Handbook of theory and research for the sociology of education.

[ref8] Bourdieu P, Richardson J (1986). The Forms of Capital. Handbook of Theory and Research for the Sociology of Education.

[ref9] Bourdieu P, Wacuant J. D (1992). An invitation to reflexive sociology.

[ref10] Baron S, Field J, Schuller T (2000). Social Capital: Critical Perspectives.

[ref11] Baum F (1999). Social capital: is it good for your health? Issues for a public health Agenda. Journal of Epidemiology and Community Health.

[ref12] Cattel V (2001). Poor people, poor places, and poor health: The mediating role of social networks and social capital. Social Forms.

[ref13] Coleman J. S (1988). Social capital in the creation of human capital. American Journal of Sociology.

[ref14] Coleman J. S (1990). Foundations of Social Theory.

[ref15] Diez-Roux A. V, Link B. G, Northridge M (2000). A multilevel analysis of income inequality and cardiovascular disease risk factors. Social Science and Medicine.

[ref16] Edmondson R (2003). Social Capital: a strategy for enhancing health?. Social Science and Medicine.

[ref17] Gooden B. I (1998). Social capital, stress, and the health of rural-African-Americans in central Virginia.

[ref18] Granovetter M. S (1985). Economic actions and social structure: The problem of embeddedness. American Journal of Sociology.

[ref19] Grossman M (1972). On the Concept of Health Capital and the Demand for Health Journal of Political Economy. http://dx.doi.org/10.1086/259880.

[ref20] Hanifan L. J (1920). The community center.

[ref21] House J. S, Willams D. R (2000). Understanding and reducing socioeconomic and racial/ethnic disparities in heath.

[ref22] Jacobs J (1961). The uses of city neighborhoods. The death and life of great American cities.

[ref23] James S. A, Schulz A. J, van Olphen J, Saegert S, Thompson J. P, Warren M. R (2001). Social capital, poverty, and community health: An exploration of linkages. Social Capital and Poor Communities.

[ref24] Kawachi I, Kennedy B.P, Grass R (1999). Social capital and self-related health: A contextual analysis. American Journal of Public Health.

[ref25] Kawachi I, Kennedy B. P, Lochner K (1997a). Long live community: Social capital as public health. The American Prospect.

[ref26] Kawachi I, Kennedy B. P, Lochner K, Prothrow-Stith D (1997b). Social capital, income inequalities, and mortality. American Journal of Public Health.

[ref27] Kawachi I, Subramanian S.V, Almeida-Filho N (2002). A glossary for health inequalities. Journal of Epidemiology and Community Health.

[ref28] Labonte R (1999). Social capital and community redevelopment: Practitioner emptor. Australian and New Zealand Journal of Public Health.

[ref29] Lin N (1999). Theory Paper ABSTRACT 102805 Building a network theory of social capital. Connections.

[ref30] Lochner K, Kawachi I, Kennedy B.P (1999). Social capital: a guide to its measurement. Health & Place.

[ref31] Loury G. C, Wallance P, LeMund A (1977). A dynamic theory of racial income differences. Women, minorities, and employment discrimination.

[ref32] Lynch J. W, Davey Smith G, Kaplan G. A, House J. S (2000). Income inequality and mortality: Importance to health of individual income, psychosocial environment, or material conditions. British Medical Journal.

[ref33] MacIntyre S, Ellaway A, Der G, Ford G, Hunt K (1998). Do housing tenure and car access predict health because they are simply markers of income or self esteem? A Scottish study. Journal of Epidemiology and Community Health.

[ref34] Marmot M, Shipley M. J, Roses M (1984). Inequalities in death-specific explanations of a general pattern?. Lancet.

[ref35] Narayan D (1997). Voices of the poor: Poverty and social capital in Tanzania.

[ref36] Paxton P (1999). Is social capital declining in the United States? A multiple indicator assessment. American Journal of Sociology.

[ref37] Portes A, Sensenbrenner J (1993). Embeddedness and immigration: Notes on the social determinants of economic action. American Journal of Sociology.

[ref38] Portes A (1998). Social Capital: Its origins and applications in modern sociology. Annual Review of Sociology.

[ref39] Preston S (1975). The Changing Relation between Mortality and Level of Development. Population Studies.

[ref40] Putnam R. D, Robert L, Rffaella Y. N (1993). Making Democracy Work.

[ref41] Putnam R. D (1995). Bowling alone: America’s declining social capital. Journal of Democracy.

[ref42] Putnam R. D (2000). Bowling alone: The collapse and revival of American community.

[ref43] Robert S. A (1999). Socioeconomic position and health: The independent contribution of socioeconomic context. Annual Review of Sociology.

[ref44] Rose R (2000). How much does social capital add to individual health? A survey study of Russians. Social Science & Medicine.

[ref45] Runyan D. K, Hunter W. M, Socolar R, Amaya-Jackson L, English D, Landsverk J, Mathew R. M (1998). Children who prosper in unfavorable environments: The relationship to social capital. Pediatrics.

[ref46] Schuller T, Baron S, Field J, Baron S, Field J, Schuller T (2000). Social capital: A review and critique. Social capital: Critical perspectives.

[ref47] Schultz T. W (1961). Investment in human capital. American Economic Review.

[ref48] Solow R. M, Dasgupta P, Serageldin I (2000). Notes on social capital and economic performance. Social capital: A multifaceted perspective.

[ref49] Soonbader M. J, LeClere F. B (1999). Aggregation and the measurement of income inequality: Effects on morbidity. Social Science & Medicine.

[ref50] Sorlie P. D, Backlund E, Keller J. B (1995). US mortality by economic, demographic, and social characteristics: The National Longitudinal Mortality Survey. American Journal of Public Health.

[ref51] Wilkinson R. G (1997). Health inequalities: Relative or absolute material standards?. British Medical Journal.

[ref52] Woolcock M (2001). The place of social capital in understanding social and economic outcomes. Isuma.

[ref53] Woolcock M (1998a). Social capital and economic development: Toward a theoretical synthesis and policy framework. Theory and Society.

[ref54] Woolcock M (1998b). Social theory, development policy, and poverty alleviation: A comparative-historical analysis of group-based banking in developing economics.

